# Explaining risk for suicidal ideation in adolescent offspring of mothers with depression

**DOI:** 10.1017/S0033291715001671

**Published:** 2015-08-25

**Authors:** G. Hammerton, S. Zammit, A. Thapar, S. Collishaw

**Affiliations:** 1 Institute of Psychological Medicine and Clinical Neurosciences, Cardiff University, Cardiff, UK; 2 Centre for Academic Mental Health, University of Bristol, Bristol, UK

**Keywords:** Adolescence, maternal depression, mechanisms, parent-child relationship, suicidal ideation

## Abstract

**Background:**

It is well-established that offspring of depressed mothers are at increased risk for suicidal ideation. However, pathways involved in the transmission of risk for suicidal ideation from depressed mothers to offspring are poorly understood. The aim of this study was to examine the contribution of potential mediators of this association, including maternal suicide attempt, offspring psychiatric disorder and the parent–child relationship.

**Method:**

Data were utilized from a population-based birth cohort (ALSPAC). Three distinct classes of maternal depression symptoms across the first 11 years of the child's life had already been identified (*minimal, moderate, chronic-severe*). Offspring suicidal ideation was assessed at age 16 years. Data were analysed using structural equation modelling.

**Results:**

There was evidence for increased risk of suicidal ideation in offspring of mothers with *chronic-severe* depression symptoms compared to offspring of mothers with *minimal* symptoms (odds ratio 3.04, 95% confidence interval 2.19–4.21). The majority of this association was explained through maternal suicide attempt and offspring psychiatric disorder. There was also evidence for an independent indirect effect via the parent–child relationship in middle childhood. There was no longer evidence of a direct effect of maternal depression on offspring suicidal ideation after accounting for all three mediators. The pattern of results was similar when examining mechanisms for maternal *moderate* depression symptoms.

**Conclusions:**

Findings highlight that suicide prevention efforts in offspring of depressed mothers should be particularly targeted at both offspring with a psychiatric disorder and offspring whose mothers have made a suicide attempt. Interventions aimed at improving the parent–child relationship may also be beneficial.

## Introduction

It is well-established that offspring of mothers with depression are at increased risk for suicide-related behaviour including ideation (Garber *et al.* 1998), suicide attempt (Lewinsohn *et al.* 2005) and suicide (Von Borczyskowski *et al.* 2011). However, the pathways that explain suicidal risk in the offspring of mothers with depression are poorly understood. The most commonly assessed explanations of increased suicidal risk are emergence of depression or other psychiatric disorder in the offspring or exposure to suicide attempts by the mother (Mittendorfer-Rutz *et al.* 2008; Gureje *et al.* 2011; Brent *et al.* 2015). However, evidence suggests that the association between maternal depression and offspring suicidal ideation is not entirely explained by offspring psychiatric disorder (Gureje *et al.* 2011) or maternal suicide attempt (Gureje *et al.* 2011; Hammerton *et al.* 2015*a*), suggesting that additional mechanisms are important to consider. However, research investigating other mediating pathways is lacking. Targeted prevention for suicidal ideation can be effective (Brent *et al.* 2013) but relies on a good understanding of mechanisms underlying the intergenerational transmission of risk, therefore establishing why offspring of depressed mothers are at increased suicide risk compared to offspring of non-depressed mothers is crucial.

Research on risk factors for adolescent suicidal ideation suggests additional possible risk pathways. A number of studies have highlighted the importance of the quality of the parent–child relationship in explaining risk for adolescent suicidal ideation (Fergusson & Lynskey, 1995; King & Merchant, 2008; Boeninger, 2013), especially lack of support or availability of family members (Thompson *et al.* 2005; Bridge *et al.* 2006; Connor & Rueter, 2006). The majority of these studies have shown that difficulties in the parent–child relationship predicts adolescent suicidal ideation independently of the adolescent's own psychopathology (Thompson *et al.* 2005; Connor & Rueter, 2006; Boeninger, 2013). Additionally, two cohort studies of depressed adolescents found that adolescent-rated poor family functioning was associated with later suicide attempt (Wilkinson *et al.* 2011; Asarnow *et al.* 2011). Previous literature has also shown that maternal depression can lead to disruptions in the parent–child relationship (Lovejoy *et al.* 2000; Keenan-Miller *et al.* 2010), and that these disruptions may be one pathway through which maternal depression increases risk for offspring psychiatric disorder (Goodman & Gotlib, 1999). However, only one study that we are aware of has tested whether the association between maternal depression and offspring suicidal ideation is explained by aspects of the family environment. Using a sample of young adolescents and their mothers (the majority of whom had a history of a mood disorder), it was found that mother and child perceptions of the family environment at baseline mediated the association between maternal history of mood disorder and offspring suicidal symptoms 1 year later (Garber *et al.* 1998).

There is considerable heterogeneity in the severity and course of adult depression, and this has not been considered in studies of mechanisms explaining offspring risk of suicide-related behaviour. Previous research does show that risk for adolescent suicide-related behaviour is not confined to families where the parent is affected by severe clinical depression, but also extends to adolescents of mothers who suffer from milder but sustained sub-threshold levels of depression (Hammerton *et al.* 2015*a*). Previous studies of risk mechanisms have focused on mothers with a lifetime diagnosis of depression (Garber *et al.* 1998; Gureje *et al.* 2011). It is important to establish if mechanisms underlying the link between maternal depression and offspring suicidal ideation in unselected population cohorts vary according to maternal depression symptom severity. In addition, very few studies have examined mediators across a longer developmental time span, covering the course of childhood and adolescence; an important consideration given that early intervention is a recognized priority in suicide prevention (National Action Alliance for Suicide Prevention, 2014). Given that a number of different inter-related pathways are likely involved in the aetiology of adolescent suicidal ideation, testing competing mechanisms together is especially important. We anticipate that these will include mothers’ own suicidal behaviour, adolescent psychiatric problems, and the relationship between at-risk children and their parents.

**Fig. 1.**
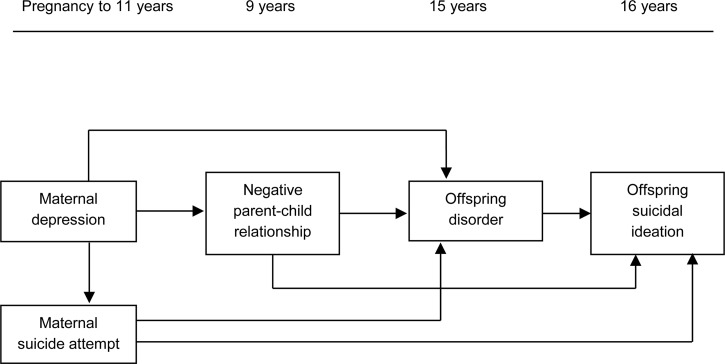
Theoretical model displaying all hypothesized pathways.

The present investigation uses a large, prospective, population cohort to examine hypothesized mediators that might contribute to the association between differing levels of maternal depression in childhood and offspring suicidal ideation in adolescence. These mediators were assessed across childhood and adolescence and include maternal suicide attempt across the first 11 years of their child's life, the parent–child relationship in middle childhood and offspring psychiatric disorder in adolescence (see Fig. 1 for the theoretical model displaying all hypothesized pathways). We hypothesize that there will be independent indirect effects via offspring psychiatric disorder, via the parent–child relationship and via maternal suicide attempt. Finally, it is expected that together these risk mediators will account for a large part of the association between maternal depression and adolescent suicidal ideation.

## Method

### Sample

Data were utilized from a large UK birth cohort study; the Avon Longitudinal Study of Parents and Children (ALSPAC). The cohort was set up to examine genetic and environmental determinants of health and development (Boyd *et al.* 2013). The core enrolled sample consisted of 14 541 pregnant women resident in the former county of Avon, UK, who had an expected date of delivery between 1 April 1991 and 31 December 1992. Of the 14 062 live births, 13 617 were singletons and were alive at age 1 year. The sample is broadly representative of the UK population; however, mothers enrolled in ALSPAC were more likely to live in owner-occupied accommodation and own a car, to be married, and were less likely to be non-white (Fraser *et al.* 2013). Parents and children have been followed up regularly since recruitment via questionnaire and clinic assessments. All adult participants gave informed consent, and ethical approval for the study was obtained from the ALSPAC Ethics and Law Committee and the Local Research Ethics Committees. Further details on the sample characteristics and methodology have been described previously (Boyd *et al.* 2013; Fraser *et al.* 2013) and detailed information about ALSPAC can be found on the study website (http://www.bristol.ac.uk/alspac). For information on all available ALSPAC data see the fully searchable data dictionary (http://www.bris.ac.uk/alspac/researchers/data-access/data-dictionary).

### Measures

#### Maternal depression symptom trajectories

Maternal depression symptoms were assessed at 10 time points (from 18 weeks gestation to child age 11 years) using the Edinburgh Postnatal Depression Scale (Cox *et al.* 1987). In a prior analysis using this dataset, latent class growth analysis was used to identify qualitatively distinct patterns of depression symptoms in mothers over time (Hammerton *et al.* 2015*a*, *b*). For the purposes of these analyses, three trajectory classes of depression were compared: mothers with chronic and severe levels of symptoms ‘chronic-severe’, those with sub-threshold but sustained depression symptoms over time ‘moderate’, and those with very low levels of depression symptoms ‘minimal’. Approximately 40% of the sample belonged to the *minimal* class, 55% of the sample belonged to the *moderate* class and 5% of the sample belonged to the *chronic-severe* class. In all analyses the *minimal* class is treated as the reference group. See Supplementary Table S1 for mean depression symptoms at each assessment for mothers in each of the groups (*minimal, moderate* and *chronic-severe*). For further details on the derivation and validation of classes see Hammerton *et al.* (2015*a, b*).

#### Offspring suicidal ideation

Suicidal ideation at age 16 years was assessed via a self-report postal questionnaire (Kidger *et al.* 2012). Participants were classified as having a lifetime history of suicidal ideation if they responded positively to either of the following questions: *Have you ever found yourself wishing you were dead and away from it all?; Have you ever thought of killing yourself, even if you would not really do it?* Participants were then asked when the last time was that they felt this way. The present investigation focuses on children who reported suicidal ideation in the previous year (78% of those who reported lifetime suicidal ideation by age 16 years) to preserve the time-ordering of the analysis.

#### Hypothesized mediating variables

*Offspring psychiatric disorder.* Offspring psychopathology was assessed at age 15 years using the Development and Well-Being Assessment (DAWBA; Goodman *et al.* 2000) parent and child versions. The DAWBA is a semi-structured interview consisting of questions about child mental health symptoms and their impact. ‘Any disorder’ [including depressive disorder, anxiety disorders, disruptive behaviour disorder (DBD), attention deficit hyperactivity disorder (ADHD) and eating disorder] was derived using a well-defined computerized algorithm that predicts the probability of a clinical rater assigning each child an ICD-10 or DSM-IV diagnosis from symptoms for each disorder (see www.DAWBA.com for more information).

*Maternal suicide attempt.* Maternal suicide attempt was assessed at 10 time points (from pregnancy to child age 11 years) using a self-report life events questionnaire (Brown & Harris, 1978) in which the mother was asked if she had attempted suicide since the previous assessment (beginning in pregnancy). All available time points were combined to create a binary ‘yes/no’ variable.

*Child perceived relationship with parents.* The parent–child relationship was assessed using nine questions to the child about their perceptions of their relationship with parents at age 9 years. The questionnaire asked the child to rate how true a number of sentences were (on a 5-point scale). Items included quality and frequency of time spent together (e.g. *my parents and I spend a lot of time together*), support (e.g. *my parents are easy to talk to*) and disapproval (e.g. *my parents are usually unhappy or disappointed with what I do*). Positive questions were reverse-coded so that, for all items, a score of 4 represented a negative parent–child relationship and items were added up to create a total score (range 0–36). A factor analysis was performed on the nine items and indicated a single factor solution (see Supplementary Table S2) and the scale showed good internal consistency (*α* = 0.79). Prior to analyses, the scale was standardized to aid interpretation.

*Potential confounders.* Potential socio-demographic and familial confounding factors assessed in pregnancy were chosen based on evidence from previous literature (Johnson *et al.* 2002; Skipstein *et al.* 2010) and associations with maternal depression symptoms and offspring suicidal ideation found in the present sample. Maternal questionnaires completed during pregnancy were used to assess housing tenure (owned *v.* rented), marital status (married *v.* single), maternal level of education (below O-level, O-level, above O-level), self-reported psychiatric disorder before pregnancy (yes/no; including drug addiction, alcoholism, schizophrenia, anorexia nervosa, severe depression or any other psychiatric disorder), maternal family history of depression (0, 1 or both parents) and smoking in pregnancy (assessed in ALSPAC as smoking tobacco in either the first 3 months or the last 2 weeks of pregnancy).

## Missing data

The starting sample for these analyses included mothers who had information on the latent classes of maternal depression symptoms (*N* = 10 559). Of the starting sample, 4588 offspring had complete data on suicidal ideation at age 16 years (43%; 1904 males and 2684 females; mean age 16.7 years, standard deviation 0.2 years) and of these, 2842 offspring also had complete data on all potential mediators (see Supplementary Fig. S1). Missing data for offspring suicidal ideation and potential mediators were imputed using multivariate imputation by chained equations (Van Buuren & Oudshoom, 2000) which assumes data are missing at random (MAR), i.e. given the observed data included in the imputation model, the missingness mechanism does not depend on the unobserved data (White *et al.* 2011). As missing data was found to be dependent on several variables, these were included in the imputation model to make the assumption of MAR as plausible as possible. In addition, the imputation model included all variables included in the analysis models, and a number of auxiliary variables that were associated with offspring suicide-related behaviour and mediators. Further detail on the imputation procedure is given in Supplement 1. The imputed sample of 10 559 is used for all analyses hereafter unless otherwise stated; however, a number of sensitivity checks were performed by repeating analyses using alternative approaches to dealing with missing data. The main tables show results using four alternative approaches: (1) full imputation, *N* = 10 559; (2) imputation for those sent questionnaires at 16, *N* = 8475; (3) imputation for those with complete outcome data, *N* = 4588; (4) complete case analysis, *N* = 2842. Supplementary Table S3 shows demographics for those with complete data (*N* = 2842) and each of the imputed samples (*N* = 4588, *N* = 8475, *N* = 10 559) in comparison to the original ALSPAC cohort that met inclusion criteria for this study (singletons and offspring alive at 1 year, *N* = 13 617). As shown in Supplementary Table S3, the imputation procedure corrected for biases present from selective attrition with the fully imputed sample being more representative of the original ALSPAC cohort than the complete case sample.

## Statistical analysis

First, univariable logistic regression analyses were performed to examine initial associations between variables. Next, a single mediation model was run using structural equation modelling (SEM) in Mplus to assess effects of *moderate* or *chronic-severe* maternal depression symptoms on offspring suicidal ideation both directly and indirectly, through offspring psychiatric disorder (at age 15 years). A weighted least squares estimator (WLSMV) was used due to its robustness in analysing both continuous and categorical measures in SEM (Muthén & Muthén, 1998–2012). Results from path analyses with a continuous outcome are presented as linear regression coefficients and results with a categorical outcome (including indirect effects) are presented as probit regression coefficients (referred to throughout as *B*). Probit coefficients refer to the strength of the association between an exposure and probability of group membership. Therefore the coefficient represents the difference that a 1-unit change in the exposure variable makes in the cumulative normal probability of the outcome variable. Indirect effects were calculated using a non-parametric bootstrapping approach with 500 replications. Next a multiple mediation model was run including the child perceived parent–child relationship at age 9 years in the model together with offspring psychiatric disorder. Finally, the full structural model was run including all mediators: offspring psychiatric disorder, the parent–child relationship and maternal suicide attempt. To examine if the indirect effects within the same model differed in strength for maternal *moderate* depression compared to maternal *chronic-severe* depression, *post-hoc* Wald χ^2^ tests were used to test the assumption of equality between constrained indirect effects. The full model was also rerun without using bootstrapping in order to calculate model fit statistics [the root-mean square error of approximation (RMSEA) and the comparative fit index (CFI)]. RMSEA values <0.05 (Browne & Cudeck, 1992) and CFI values >0.90 (Hu *et al.* 1992) indicate close fit. Finally, subgroup comparisons using stacked modelling procedures (Bollen, 1989) were used to assess whether the magnitude of parameter estimates differed in strength for males and females. *Post-hoc* Wald *χ*^2^ tests were used to test the assumption of equality between the targeted paths (indirect effects of maternal *chronic-severe* depression on offspring suicidal ideation) across gender. Analyses were conducted using Stata v. 13 (StataCorp, 2013) and Mplus v. 7 (Muthén & Muthén, 1998–2012).

## Results

Fifteen percent of adolescents [95% confidence interval (CI) 14–17; 11% of males, 20% of females] reported past year suicidal ideation at age 16 years’ assessment† and 9% of adolescents (95% CI 8–10) met DSM-IV or ICD-10 criteria for ‘any disorder’ (including depressive disorder, anxiety disorders, DBD, ADHD or eating disorder) at 15 years. Two percent of mothers made a suicide attempt between pregnancy and child age 11 years.

Table 1 shows an increase in offspring suicidal ideation, offspring psychiatric disorder, maternal suicide attempt and parent–child relationship difficulties with increasing severity of maternal depression symptoms. Odds of suicidal ideation and psychiatric disorder were elevated approximately 2- to 6-fold in moderate and severely depressed risk groups.

**Table 1.** Pattern of maternal suicide attempt, offspring psychiatric disorder at age 15 years, offspring suicidal ideation at age 16 years and parent–child relationship at age 9 years by classes of maternal depression symptoms (imputed N = 10 559)

				Moderate *v.* minimal	Chronic-severe *v.* minimal
	Minimal	Moderate	Chronic-severe	OR/*β* (95% CI)	OR/*β* (95% CI)
Offspring suicidal ideation (%)	11.74	16.69	28.81	1.51 (1.30–1.75)*	3.04 (2.19–4.21)*
Offspring psychiatric disorder (%)	4.89	10.31	22.10	2.24 (1.79–2.80)*	5.51 (3.92–7.74)*
Maternal suicide attempt (%)	0.34	2.32	10.87	7.05 (4.06–12.25)*	36.26 (20.12–65.37)*
Parent–child relationship (mean)	2.94	3.46	4.15	0.14 (0.09–0.19)*	0.26 (0.15–0.37)*

OR, Odds ratio; *β*, beta coefficient; CI, confidence interval

*p < 0.001.

### Mediation of effect of maternal depression on offspring suicidal ideation

#### Offspring psychiatric disorder

Supplementary Fig. S2 shows results from the structural model examining the direct effect of maternal *chronic-severe* depression (with *minimal* class as the reference group), on offspring past year suicidal ideation at age 16 years, and the indirect effect through offspring disorder at age 15 years. There was evidence of an indirect effect through offspring disorder [*B* (95% CI) = 0.35 (0.24–0.46)] and a direct effect not mediated by offspring disorder [*B* (95% CI) = 0.28 (0.07–0.48), *p* = 0.007].

A similar pattern of results was found for offspring of mothers with *moderate* depression symptoms over time (in comparison to offspring of mothers with *minimal* symptoms) with evidence of an indirect effect through offspring disorder [*B* (95% CI) = 0.16 (0.10–0.21)]. However, there was no evidence of a direct effect of maternal *moderate* symptoms on offspring suicidal ideation after accounting for offspring disorder [*B* (95% CI) = 0.07 (−0.03 to 0.16); *p* = 0.161].

#### Offspring disorder and child perceived parent–child relationship

Supplementary Fig. S3 shows results from the structural model examining the direct effect of maternal *chronic-severe* depression on offspring suicidal ideation, and the indirect effects through the parent–child relationship at age 9 years and offspring psychiatric disorder at age 15 years. There was evidence of indirect effects via only the parent–child relationship [*B* (95% CI) = 0.03 (0.01–0.06)], via offspring disorder alone [*B* (95% CI) = 0.36 (0.23–0.49)], and via both parent–child relationship and offspring disorder [*B* (95% CI) = 0.01 (0.003–0.02)]. A similar pattern of results was found for offspring of mothers with *moderate* symptoms over time (in comparison to offspring of mothers with *minimal* symptoms) with evidence of an indirect effect via only the parent–child relationship [*B* (95% CI) = 0.02 (0.01–0.03)], via offspring disorder alone [*B* (95% CI) = 0.16 (0.10–0.22)], and via both parent–child relationship and offspring disorder [*B* (95% CI) = 0.01 (0.002–0.01)].

#### Offspring disorder, parent–child relationship and maternal suicide attempt

**Fig. 2.**
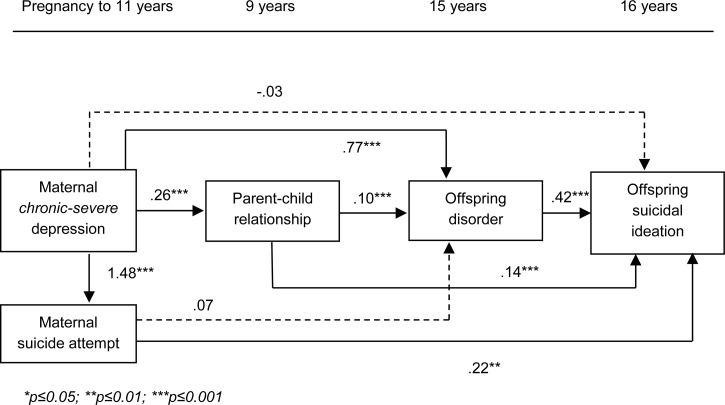
Full structural model showing the direct effect of maternal chronic-severe depression (with minimal class as the reference group) on offspring past year suicidal ideation at age 16 years, and the indirect effects through offspring psychiatric disorder, the parent–child relationship and maternal suicide attempt; imputed *N* = 10 559; non-standardized probit regression coefficients presented for categorical outcomes; linear regression coefficient presented for continuous outcome (parent–child relationship).

Fig. 2 shows results from the full structural model examining the direct effect of maternal *chronic-severe* depression on offspring suicidal ideation, and the indirect effects through offspring disorder, the parent–child relationship and maternal suicide attempt. There was no longer evidence of a direct effect of maternal *chronic-severe* depression on offspring suicidal ideation after accounting for all potential mediators [*B* (95% CI) = −0.03 (−0.32 to 0.26); *p* = 0.852]. Model fit statistics indicated a good fit to the data (RMSEA = 0.003; CFI = 1.000).

Table 2 (model 1a) shows the unadjusted indirect effects through all possible combinations of potential mediators. There was evidence of an indirect effect via maternal suicide attempt [*B* (95% CI) = 0.33 (0.11–0.54)], via offspring disorder [*B* (95% CI) = 0.32 (0.16–0.48)], via the parent–child relationship [*B* (95% CI) = 0.04 (0.01–0.06)] and via both the parent–child relationship and offspring disorder [*B* (95% CI) = 0.01 (0.003–0.02)]. Of the total effect [*B* (95% CI) = 0.71 (0.49–0.93], 46% was explained through maternal suicide attempt, 45% was explained through offspring disorder, 6% was explained through the parent–child relationship and 1% was explained through both the parent–child relationship and offspring disorder. Table 2 (model 1b) shows that the pattern of results was the same after adjusting for potential confounders assessed in pregnancy (child gender, housing tenure, marital status, maternal level of education, smoking in pregnancy, maternal family history of depression and maternal psychiatric disorder before pregnancy).

**Table 2.** Indirect effect of maternal chronic-severe depression (with minimal class as the reference group) on offspring suicidal ideation through all possible combinations of mediators (non-standardized probit regression coefficient and 95% confidence intervals displayed)

	Indirect effects of maternal chronic-severe depression on suicidal ideation via mediators				
	[probit coefficient (95% confidence interval)]				
Model^a^	Offspring disorder	Parent–child (PC) relationship	PC relationship and offspring disorder	Maternal suicide attempt	Maternal suicide attempt and offspring disorder
Model 1a: using full imputed data; unadjusted (*N* = 10 559)	0.32 (0.16 to 0.48)	0.04 (0.01–0.06)	0.01 (0.003–0.02)	0.33 (0.11 to 0.54)	0.04 (−0.05 to 0.14)
Model 1b: adjusted for confounders (*N* = 10 559)^b^	0.27 (0.12 to 0.42)	0.04 (0.02–0.07)	0.01 (0.004–0.02)	0.26 (0.07 to 0.45)	−0.01 (−0.09 to 0.07)
Model 2: as model 1a, imputing those that were sent questionnaire (*N* = 8475)	0.30 (0.13 to 0.46)	0.03 (0.01–0.06)	0.01 (0.002–0.02)	0.33 (0.08 to 0.58)	0.02 (−0.03 to 0.07)
Model 3: as model 1a, imputing those with complete outcome data (*N* = 4588)	0.22 (−0.001 to 0.44)	0.03 (0.01–0.06)	0.01 (0.000–0.03)	0.28 (−0.03 to 0.59)	0.06 (−0.10 to 0.21)
Model 4: as model 1a, complete cases (*N* = 2842)	0.18 (0.01 to 0.45)	0.05 (0.02–0.10)	0.01 (0.004–0.04)	0.19 (−0.14 to 0.63)	0.05 (−0.11 to 0.19)

a Model 1a shows the unadjusted results using the full imputed dataset; model 1b shows results after adjusting for confounders assessed in pregnancy; model 2 shows the unadjusted results using imputed data for those offspring that were sent the questionnaire at age 16 years; model 3 shows the unadjusted results using imputed data for mediators in those that had complete outcome data; model 4 shows the unadjusted results using only those with complete data on all variables in analysis.

b Adjusting for confounders assessed in pregnancy (child gender, housing tenure, marital status, maternal level of education, smoking in pregnancy, maternal family history of depression and maternal psychiatric disorder before pregnancy).

Sensitivity checks were performed by rerunning the full model using alternative approaches to dealing with missing data. Findings were comparable when only imputing data for those offspring that were sent the questionnaire measure at age 16 years (*N* = 8475; Table 2, model 2) and when only imputing mediators for those offspring with complete outcome data (*N* = 4588; Table 2, model 3). Finally, although indirect effects were generally weaker when using complete cases (*N* = 2842; Table 2, model 4), conclusions remained unchanged with the exception of the indirect effect through maternal suicide attempt (due to a weaker association between maternal suicide attempt and offspring suicidal ideation in complete case analyses; not shown).

The pattern of results was similar when examining indirect effects of maternal *moderate* depression symptoms on offspring suicidal ideation through offspring disorder, the parent–child relationship and maternal suicide attempt (Table 3, model 1a). There was evidence of an indirect effect via maternal suicide attempt [*B* (95% CI) = 0.16 (0.05–0.27)], via offspring disorder [*B* (95% CI) = 0.14 (0.06–0.22)], via the parent–child relationship [*B* (95% CI) = 0.02 (0.01–0.03)] and via both the parent–child relationship and offspring disorder [*B* (95% CI) = 0.01 (0.002–0.01)]. Again, the pattern of results was the same after adjusting for potential confounders (Table 3, model 1b). Findings were comparable across different imputation samples (Table 3, models 2 and 3); however, all indirect effects were weaker when using complete cases (Table 3, model 4). Next, *post-hoc* Wald *χ*^2^ tests were performed to compare the strength of parameters for each indirect effect of maternal *moderate* depression on offspring suicidal ideation to the same indirect effect for maternal *chronic-severe* depression. There was evidence that the indirect effects via maternal suicide attempt, via offspring disorder and via the parent–child relationship were each stronger for maternal *chronic-severe* depression compared to the same indirect effect for maternal *moderate* depression (all *p* < 0.037).

**Table 3.** Indirect effect of maternal moderate depression (with minimal class as the reference group) on offspring suicidal ideation through all possible combinations of mediators (non-standardized probit regression coefficient and 95% confidence intervals displayed)

	Indirect effects of maternal moderate depression on suicidal ideation via mediators [probit coefficient (95% CI)]				
Model^a^	Offspring disorder	Parent–child (PC) relationship	PC relationship and offspring disorder	Maternal suicide attempt	Maternal suicide attempt and offspring disorder
Model 1a: using full imputed data; unadjusted (*N* = 10 559)	0.14 (0.06 to 0.22)	0.02 (0.01–0.03)	0.01 (0.002 to 0.01)	0.16 (0.05 to 0.27)	0.02 (−0.03 to 0.07)
Model 1b: adjusted for confounders (*N* = 10 559)^b^	0.12 (0.05 to 0.20)	0.02 (0.01–0.03)	0.01 (0.002 to 0.01)	0.13 (0.03 to 0.23)	−0.01 (−0.05 to 0.04)
Model 2: as model 1a, imputing those that were sent questionnaire (*N* = 8475)	0.13 (0.05 to 0.22)	0.02 (0.01–0.03)	0.01 (0.001 to 0.01)	0.17 (0.04 to 0.30)	0.01 (−0.04 to 0.06)
Model 3: as model 1a, imputing those with complete outcome data (*N* = 4588)	0.10 (−0.01 to 0.21)	0.01 (0.002–0.02)	0.01 (−0.001 to 0.01)	0.13 (−0.02 to 0.29)	0.03 (−0.05 to 0.10)
Model 4: as model 1a, complete cases (*N* = 2842)	0.07 (−0.04 to 0.20)	0.01 (0.003–0.03)	0.004 (0.001 to 0.01)	0.11 (−0.09 to 0.37)	0.03 (−0.06 to 0.12)

a Model 1a shows the unadjusted results using the full imputed dataset; model 1b shows results after adjusting for confounders assessed in pregnancy; model 2 shows the unadjusted results using imputed data for those offspring that were sent the questionnaire at age 16 years; model 3 shows the unadjusted results using imputed data for mediators in those that had complete outcome data; model 4 shows the unadjusted results using only those with complete data on all variables in analysis.

b Adjusting for confounders assessed in pregnancy (child gender, housing tenure, marital status, maternal level of education, smoking in pregnancy, maternal family history of depression and maternal psychiatric disorder before pregnancy).

Main analyses were then run separately by gender. Supplementary Fig. S4(*a, b*) show results from the structural models examining the direct effect of maternal *chronic-severe* depression on offspring suicidal ideation, and the indirect effects through the parent–child relationship, offspring psychiatric disorder and maternal suicide attempt for males and females respectively. *Post-hoc* Wald χ^2^ tests were performed to compare the strength of parameters for each indirect effect of maternal *chronic-severe* depression on offspring suicidal ideation by gender. There was evidence that the indirect effect via offspring psychiatric disorder was stronger in females compared to males (*p* = 0.015).

Finally, all results were replicated when examining the impact of maternal depression symptoms from pregnancy to child age 8 years to preserve the time-ordering of measures (results available from first author).

## Discussion

Consistent with previous research (Garber *et al.* 1998; Gureje *et al.* 2011), offspring in this unselected population cohort that were exposed to chronic and severe maternal depression symptoms were at considerably increased risk for later suicidal ideation in adolescence. Although the majority of the association between maternal *chronic-severe* depression and offspring suicidal ideation was explained through maternal suicide attempt and offspring psychiatric disorder, there was also evidence for an independent indirect effect via the parent–child relationship in middle childhood. As hypothesized, there was no longer evidence of a direct effect of maternal *chronic-severe* depression on offspring suicidal ideation after accounting for all three mediators. Maternal *moderate* depression symptoms were considerably more common affecting half of children in this cohort. These offspring were also at increased risk for suicidal ideation, highlighting the importance of considering suicidal ideation and related risk pathways in mothers with sub-threshold but sustained symptoms of depression. Findings showed that the same mechanisms accounted for risks in this group. Again, maternal suicide attempt, offspring psychiatric disorder and problems in the parent–child relationship together fully accounted for the association between maternal *moderate* depression and adolescent suicidal ideation.

In this population sample, offspring proximal psychiatric disorder and maternal suicide attempt explained the majority of the association between maternal depression and offspring suicidal ideation. This supports previous studies that have highlighted the importance of both these mechanisms (Mittendorfer-Rutz *et al.* 2008; Gureje *et al.* 2011; Geulayov *et al.* 2014). Few studies however, have examined other potential mechanisms of the association between maternal depression and offspring suicidal ideation. The direct association between the parent–child relationship and offspring suicidal ideation found in this study supports previous literature that has shown that disruption to the parent–child relationship predicts suicidal ideation independently to the adolescent's own psychopathology (Thompson *et al.* 2005; Connor & Rueter, 2006; Boeninger, 2013). The current study extends this research by demonstrating a small, yet robust indirect effect of maternal depression on offspring suicidal ideation via the child perceived parent–child relationship in middle childhood that was not fully accounted for by offspring psychiatric disorder or potential confounding factors. The same mechanisms were found for offspring of mothers with sub-threshold levels of depression symptoms over time. This finding builds on the previous study that identified family functioning as a mediator of the association between maternal history of mood disorder and offspring suicidal ideation (Garber *et al.* 1998) by showing that the parent–child relationship is also an important mechanism for offspring of mothers with less severe levels of depression symptoms that may have never been diagnosed with a mood disorder.

In the current study, the effect of the parent–child relationship on later suicidal ideation was explained, in part, through the presence of a psychiatric disorder in the offspring. The remaining direct effect of the parent–child relationship on suicidal ideation could be explained by a number of factors. A negative parent–child relationship may lead the child to feel like an expendable member of the family (Van Orden *et al.* 2010); alternatively lack of support and communication with parents may lead the child to feel that thoughts of suicide are the only method of escape (Garber *et al.* 1998). Additionally, a recent study that utilized the same sample (ALSPAC) found that the association between an adverse early family environment and self-harm in adolescence was partially mediated by peer victimisation (Lereya *et al.* 2013). Finally, although most associations appeared weaker for males compared to females, there was no evidence that the indirect effects differed across gender with the exception of the indirect effect of maternal depression on offspring suicidal ideation via offspring psychiatric disorder which was stronger for females. These findings are in line with previous literature that has reported that familial transmission of psychopathology is stronger in parents and children of the same sex (Lewis *et al.* 2011; Goodman *et al.* 2011). However, more research is needed to replicate the gender differences observed here.

The findings need to be considered in the light of several limitations. First, as the parent–child relationship and suicidal outcome measure were both reported by the child, shared rater bias may have inflated associations with offspring suicidal ideation (even though reported 7 years apart). However, it is widely agreed that children's own perceptions of relationships are particularly important when considering risk for psychopathology and suicidal ideation. Second, as with most cohort studies, there was selective attrition over time, and only a minority of cohort members provided data on all measures across childhood and adolescence; however, potential bias arising from missing data was dealt with using multiple imputation, utilizing a large amount of additional information to make the assumption of missing-at-random as plausible as possible. Indeed missing data biases were accounted for well in imputed models. Additionally, findings were comparable across three different samples of imputed data. Although most indirect effects were weaker in complete case analyses, as has been reported previously (Pearson *et al.* 2013; Hammerton *et al.* 2015*b*), the pattern of findings was similar (with the exception of a weaker association between maternal suicide attempt and offspring suicidal ideation in those with complete data). Third, although this study allowed for the time-ordering of effects to be examined, it is still important to consider the possibility of reverse causation as the direction of effects between the potential mediators examined is not well-established. It is also possible that earlier suicidal behaviour impacted on offspring psychiatric disorder as has been reported previously in the same sample (Mars *et al.* 2014). Finally, although this study assessed a number of competing mechanisms of the association between maternal depression and offspring suicidal ideation, there are additional mechanisms that may also contribute that were not assessed here including peer victimization, offspring temperament and personality and cognitive behavioural factors such as coping skills. Additionally, other aspects of the family environment will be important for future research to consider including parental discord, family structure and sibling relationships.

The current study suggests that parent, child and family-related mechanisms explain the association between maternal depression and offspring suicidal ideation. These findings have implications for future theory development and model testing. Genetically sensitive designs such as those involving children-of-twins (Silberg *et al.* 2010), children born by assisted conception (Thapar *et al.* 2007) and adoption studies (Elam *et al.* 2014) will be especially helpful in replicating these observational findings because they can take into account genetic contributors to cross-generational associations (Thapar & Rutter, 2015). As expected, offspring with a psychiatric disorder or whose mothers had made a suicide attempt were most at risk for future suicidal ideation indicating that suicide prevention efforts in offspring of depressed mothers should be targeted at these subgroups. However, there was also evidence for an additional and independent pathway from maternal depression to offspring suicidal ideation via the parent–child relationship in middle childhood. This is an important finding given that the parent–child relationship is potentially modifiable (Scott & Gardener, 2015) and therefore could be a focus of preventive interventions. A recent review highlighted that successful interventions aimed at reducing suicidal ideation and suicide attempt in adolescents often had a focus on family interactions (Brent *et al.* 2013). Therefore interventions in offspring of depressed mothers aimed at improving support and communication between mother and child as well as treating child psychopathology may also be beneficial in reducing adolescent suicide risk within this high-risk group. Given the long term negative consequences of adolescent suicidal ideation (Reinherz *et al.* 2006), targeting these children early, before the onset of suicidal behaviour, is likely to be valuable. Results also generalized to offspring of mothers with less severe levels of depression symptoms. These offspring are an important group to consider as they may be less likely to be known to services if mothers have never been diagnosed with clinical depression.

## Footnotes

† Prevalence reported for imputed datasets; for complete cases, 672/4588 (15%; 174 males, 498 females) adolescents reported past year suicidal ideation at the age 16 years’ assessment.

## Supplementary material

For supplementary material accompanying this paper visit http://dx.doi.org/10.1017//S0033291715001671. click here to view supplementary material
