# Supplementary material for: Explaining risk for suicidal ideation in adolescent offspring of mothers with depression
Source: Psychol Med. 2015 Aug 25;46(2):265–75. doi: 10.1017/S0033291715001671 (PMC4682478; doi:10.1017/S0033291715001671)
Supplement: Supplementary file 1 [file S0033291715001671sup001.zip › Hammerton_Supplement 1_revised.docx]

Supplement 1 - *Missing data imputation using Multivariate Imputation by Chained Equations*

Missing data were imputed with a fully conditional specification using the MICE (White et al. 2011) algorithm in STATA 13. As the ALSPAC sample has substantial information on socio-demographic variables that predict missingness, missing information can be assumed to be dependent on observed data. Those missing information on outcome and mediators differed from the starting sample on a number of demographic characteristics (including younger maternal age, increased smoking in pregnancy, increased parity, higher crowding index, lower maternal education and lower socio-economic status among others). Therefore, these variables were included in the imputation model to make the assumption of MAR as plausible as possible. The imputation model also included other measures that have been found to be closely associated with offspring suicidal ideation and psychiatric disorder (including measures of offspring suicidal ideation, self-harm, psychiatric disorder and symptoms at multiple time points), and the parent-child relationship (including earlier measures of parenting) and all other variables included in the analyses (full list available on request) (White et al. 2011). Using binary and ordinal logistic and linear regression models as appropriate, 80 imputed datasets were derived, each with 10 cycles of regression switching. Predictive mean matching was used when continuous variables were not normally distributed. Where analyses were run separately in males and females, data were imputed separately by gender. All analyses were then run on imputed datasets by combining estimates using Rubin’s rules (White et al. 2011). FMI values were found to be no larger than 0.7 and the Monte-Carlo errors of coefficients were approximately 10% of the standard error (White et al. 2011).
