# Supplementary material for: Explaining risk for suicidal ideation in adolescent offspring of mothers with depression
Source: Psychol Med. 2015 Aug 25;46(2):265–75. doi: 10.1017/S0033291715001671 (PMC4682478; doi:10.1017/S0033291715001671)
Supplement: Supplementary file 1 [file S0033291715001671sup001.zip › Hammerton_Supplementary Figure 1_revised.docx]

**Pregnant women enrolled in ALSPAC Phase 1**

*N* = 14,541

**Exclusion criteria**

Multiple births

Children not alive at 1 year

**Initial sample after exclusions**

*N* = 13,617

**Excluded because:**

Mothers completed less than half of EPDS assessments (*N* = 3,058)

**Sample used to derive latent classes of maternal depression symptoms**

*N* = 10,559

**Offspring received age 16 questionnaire**

*N* = 8,475

**Missing data** *(total N* = 3,887) **because:**

Did not return questionnaire

(*N* =3,820)

Did not complete questions on suicidal ideation (*N* = 67)

**Complete data for suicidal ideation at age 16 years**

*N* = 4,588

**Missing data because:**

Missing information on potential mediators (*N* = 1,746)

**Information available for all variables in main analyses**

*N* = 2,842

Supplementary Figure S1 *– Flow chart of retention in the ‘Avon Longitudinal Study of Parents and Children’ (ALSPAC) sample*
