# Supplementary material for: Explaining risk for suicidal ideation in adolescent offspring of mothers with depression
Source: Psychol Med. 2015 Aug 25;46(2):265–75. doi: 10.1017/S0033291715001671 (PMC4682478; doi:10.1017/S0033291715001671)
Supplement: Supplementary file 1 [file S0033291715001671sup001.zip › Hammerton_Supplementary Figure 2_revised.docx]

Pregnancy to 11 years 15 years 16 years

.28**

Maternal *chronic-severe* depression

Offspring suicidal ideation

Offspring disorder

.40***

.89***

**p≤0.05; **p≤0.01; ***p≤0.001*

**Supplementary Figure S2** – *Structural model showing the direct effect of maternal chronic-severe depression (with minimal class as the reference group) on offspring past year suicidal ideation at age 16 years, and the indirect effect through offspring psychiatric disorder at age 15 years; imputed N=10,559; non-standardised probit regression coefficients presented*
