# Supplementary material for: Explaining risk for suicidal ideation in adolescent offspring of mothers with depression
Source: Psychol Med. 2015 Aug 25;46(2):265–75. doi: 10.1017/S0033291715001671 (PMC4682478; doi:10.1017/S0033291715001671)
Supplement: Supplementary file 1 [file S0033291715001671sup001.zip › Hammerton_Supplementary Figure 3_revised.docx]

Pregnancy to 11 years 9 years 15 years 16 years

.28*

Offspring suicidal ideation

.86***

Maternal *chronic-severe* depression

.42***

.10***

.26***

Parent-child relationship

Offspring disorder

.13***

**p≤0.05; **p≤0.01; ***p≤0.001*

**Supplementary Figure S3** – *Structural model showing the direct effect of maternal chronic-severe depression (with minimal class as the reference group) on offspring past year suicidal ideation at age 16 years, and the indirect effect through parent-child relationship at age 9 years and offspring psychiatric disorder at age 15 years; imputed N=10,559; non-standardised probit regression coefficients presented for categorical outcomes; linear regression coefficient presented for continuous outcome (parent-child relationship)*
