# Supplementary material for: Explaining risk for suicidal ideation in adolescent offspring of mothers with depression
Source: Psychol Med. 2015 Aug 25;46(2):265–75. doi: 10.1017/S0033291715001671 (PMC4682478; doi:10.1017/S0033291715001671)
Supplement: Supplementary file 1 [file S0033291715001671sup001.zip › Hammerton_Supplementary Figure 4.docx]

Pregnancy to 11 years 9 years 15 years 16 years

.35

Offspring suicidal ideation

.49*

Maternal *chronic-severe* depression

.17^#^

.13*

.14

Parent-child relationship

Offspring disorder

1.62***

.23***

.14

Maternal suicide attempt

.16

^#^*p≤0.10; *p≤0.05; **p≤0.01; ***p≤0.001*

**Supplementary Figure S4a** – *Full* s*tructural model showing the direct effect of maternal chronic-severe depression (with minimal class as the reference group) on offspring past year suicidal ideation at age 16 years, and the indirect effects through offspring psychiatric disorder, the parent-child relationship and maternal suicide attempt in males; imputed N = 5,453; non-standardised probit regression coefficients presented for categorical outcomes; linear regression coefficient presented for continuous outcome (parent-child relationship)*

Pregnancy to 11 years 9 years 15 years 16 years

-.24

Offspring suicidal ideation

.91***

Maternal *chronic-severe* depression

.61***

.06

.39***

Parent-child relationship

Offspring disorder

1.36***

.17***

.02

Maternal suicide attempt

.27*

^#^*p≤0.10; *p≤0.05; **p≤0.01; ***p≤0.001*

**Supplementary Figure S4b** – *Full* s*tructural model showing the direct effect of maternal chronic-severe depression (with minimal class as the reference group) on offspring past year suicidal ideation at age 16 years, and the indirect effects through offspring psychiatric disorder, the parent-child relationship and maternal suicide attempt in females; imputed N = 5,106; non-standardised probit regression coefficients presented for categorical outcomes; linear regression coefficient presented for continuous outcome (parent-child relationship)*
