# Supplementary material for: Explaining risk for suicidal ideation in adolescent offspring of mothers with depression
Source: Psychol Med. 2015 Aug 25;46(2):265–75. doi: 10.1017/S0033291715001671 (PMC4682478; doi:10.1017/S0033291715001671)
Supplement: Supplementary file 1 [file S0033291715001671sup001.zip › Hammerton_Supplementary Table 1.docx]

Supplementary Table S1 **-** *Mean depression symptoms (with 95% Confidence Intervals (95% CI)) at each assessment for mothers in the chronic-severe class, moderate class and minimal class; N=10,559; clinical cut-off on EPDS=13*

| Child age at EPDS assessment | Mean (95% CI) | | |
| --- | --- | --- | --- |
|  | Chronic-severe class (5.2%) | Moderate class (55.2%) | Minimal class (39.6%) |
| 18 weeks gest | 14.3 (13.9, 14.7) | 8.4 (8.3, 8.5) | 3.5 (3.4, 3.6) |
| 32 weeks gest | 15.5 (15.1, 15.9) | 8.7 (8.6, 8.8) | 3.3 (3.2, 3.4) |
| 8 weeks | 14.8 (14.4, 15.2) | 7.5 (7.4, 7.6) | 2.6 (2.5, 2.7) |
| 8 months | 14.7 (14.3, 15.1) | 6.9 (6.8, 7.0) | 2.0 (2.0, 2.1) |
| 21 months | 15.5 (15.2, 15.9) | 7.3 (7.2, 7.4) | 2.3 (2.2, 2.4) |
| 33 months | 15.7 (15.3, 16.1) | 8.0 (7.9, 8.2) | 2.6 (2.6, 2.7) |
| 61 months | 15.4 (15.0, 15.9) | 7.7 (7.6, 7.8) | 2.6 (2.5, 2.6) |
| 73 months | 15.6 (15.2, 16.1) | 8.1 (7.9, 8.2) | 2.8 (2.7, 2.9) |
| 97 months | 15.6 (15.0, 16.1) | 7.7 (7.6, 7.8) | 2.7 (2.6, 2.8) |
| 134 months | 15.2 (14.7, 15.8) | 7.4 (7.2, 7.5) | 2.6 (2.5, 2.8) |
