# Supplementary material for: Explaining risk for suicidal ideation in adolescent offspring of mothers with depression
Source: Psychol Med. 2015 Aug 25;46(2):265–75. doi: 10.1017/S0033291715001671 (PMC4682478; doi:10.1017/S0033291715001671)
Supplement: Supplementary file 1 [file S0033291715001671sup001.zip › Hammerton_Supplementary Table 2.docx]

Supplementary Table S2 **–** *Factor analysis of nine items assessing parent-child relationship at age 9 years*

| Question | Factor loadings |
| --- | --- |
| 1. My parents understand me | 0.58 |
| 2. My parents are usually unhappy or disappointed with what I do | -0.37 |
| 3. I like my parents | 0.54 |
| 4. My parents like me | 0.50 |
| 5. If I have children of my own, I want to bring them up like my parents have brought me up | 0.42 |
| 6. My parents and I spend a lot of time together | 0.59 |
| 7. My parents are easy to talk to | 0.68 |
| 8. I get along well with my parents | 0.73 |
| 9. My parents and I have a lot of fun together | 0.68 |
| Eigenvalue | 2.99 |
