# Supplementary material for: Explaining risk for suicidal ideation in adolescent offspring of mothers with depression
Source: Psychol Med. 2015 Aug 25;46(2):265–75. doi: 10.1017/S0033291715001671 (PMC4682478; doi:10.1017/S0033291715001671)
Supplement: Supplementary file 1 [file S0033291715001671sup001.zip › Hammerton_Supplementary Table 3.docx]

Supplementary Table S3 – *Demographics of the complete case sample, three imputed samples and the original cohort that met inclusion criteria*

| *Sample demographics assessed during pregnancy^1^* | Complete cases ^a^  *(N ≤ 2,842)* | Imputed sample ^b^  *(N = 4,588)* | Imputed sample ^c^  *(N =* 8,475*)* | Imputed sample ^d^  *(N = 10,559)* | Initial cohort ^e^  *(N ≤ 13617)* |
| --- | --- | --- | --- | --- | --- |
| Female offspring *(%)* | 56.9 | 58.5 | 50.5 | 48.4 | 48.4 |
| Smoked in pregnancy *(%)* | 13.6 | 16.6 | 20.7 | 22.5 | 25.8 |
| Housing tenure *(% rented)* | 11.8 | 14.5 | 19.1 | 21.5 | 26.7 |
| Marital status *(% single)* | 15.3 | 17.0 | 19.8 | 21.5 | 23.5 |
| Maternal education *(% < O-level)* | 15.3 | 18.4 | 24.2 | 26.6 | 26.7 |
| Maternal depression *(mean EPDS score at 32 weeks gestation)* | 6.20 | 6.36 | 6.77 | 6.90 | 7.05 |

*^a^ Sample with complete data on maternal depression classes, offspring suicidal ideation and mediators; ^b^ Sample with imputed data for mediators and confounders in those that had complete outcome data; ^c^ Sample with imputed data for mediators, confounders and outcome in those offspring that were sent the questionnaire at age 16 years; ^d^ Sample with imputed data for mediators, confounders and outcome in those that had complete exposure data (for those that have information on latent classes of maternal depression symptoms; main sample used throughout results section of manuscript); ^c^Original ALSPAC cohort that the met inclusion criteria for this study*

*^1^ Additional missing data on demographics: smoked in pregnancy missing for 792/13617; housing tenure missing for 914/13617; marital status missing for 858/13617; maternal education missing for1515 /13617; maternal Edinburgh Postnatal Depression Scale (EPDS) missing for 1895/13617*
